# Evaluation of A Novel Split-Feeding Anaerobic/Oxic Baffled Reactor (A/OBR) For Foodwaste Anaerobic Digestate: Performance, Modeling and Bacterial Community

**DOI:** 10.1038/srep34640

**Published:** 2016-10-06

**Authors:** Shaojie Wang, Liyu Peng, Yixin Jiang, Petros Gikas, Baoning Zhu, Haijia Su

**Affiliations:** 1 Beijing Key Laboratory of Chemical Resource Engineering, Beijing University of Chemical Technology, Beijing, 100029, PR China; 2 School of Environmental Engineering, Technical University of Crete, 73100, Chania, Greece; 3 Beijing Higher Institution Engineering Research Center of Environmental Pollution Control and Resource Utilization, Beijing University of Chemical Technology, No. 15 Beisanhuan East Road, Chaoyang District, Beijing 100029, PR China

## Abstract

To enhance the treatment efficiency from an anaerobic digester, a novel six-compartment anaerobic/oxic baffled reactor (A/OBR) was employed. Two kinds of split-feeding A/OBRs R2 and R3, with influent fed in the 1^st^, 3^rd^ and 5^th^ compartment of the reactor simultaneously at the respective ratios of 6:3:1 and 6:2:2, were compared with the regular-feeding reactor R1 when all influent was fed in the 1^st^ compartment (control). Three aspects, the COD removal, the hydraulic characteristics and the bacterial community, were systematically investigated, compared and evaluated. The results indicated that R2 and R3 had similar tolerance to loading shock, but the R2 had the highest COD removal of 91.6% with a final effluent of 345 mg/L. The mixing patterns in both split-feeding reactors were intermediate between plug-flow and completely-mixed, with dead spaces between 8.17% and 8.35% compared with a 31.9% dead space in R1. Polymerase chain reaction-denaturing gradient gel electrophoresis (PCR-DGGE) analysis revealed that the split-feeding strategy provided a higher bacterial diversity and more stable bacterial community than that in the regular-feeding strategy. Further analysis indicated that *Firmicutes*, *Bacteroidetes*, and *Proteobacteria* were the dominant bacteria, among which *Firmicutes* and *Bacteroidetes* might be responsible for organic matter degradation and *Proteobacteria* for nitrification and denitrification.

Food waste, which is generated by the feedstock sorting, peeling, cooking and dining processes, usually accounts for 30% (w/w) of organics^1^. As a crowded capital of more than 21 million residents, Beijing has a daily food waste generation of more than 2000 t/d^2^. Traditional solid waste disposal technologies, such as landfill, composting and incineration, are mostly incapable of treating food waste because the waste has high contents of water and biodegradable organics^3^. Anaerobic digestion has been claimed as an alternative technology in China to dispose of food waste and produce renewable biogas energy using a continuous stirred tank reactor (CSTR)^1^. However, only around 40% of the total solids in food waste can be degraded. In addition, a large amount of extra water has to be added into this system to maintain a constant water content, resulting in a huge amount of digestate. Therefore, the digestate is necessary to be treated before it is discharged into the environment^1^,^4^,^5^.

Various studies have been conducted on the biological treatment of sewage sludge to reduce organic contents, but few focused on the treatment of anaerobic digestate. This is mainly because that digestate consists of high contents of salts with less content of nutrients (N, P and K), which are hard to be removed. The selection of suitable technologies has become a critical issue for the practical application in the digestate treatment. An increasing number of studies has focused on the integration of the aerobic and anaerobic process to enhance the degradation ability of the reactor. Their results suggested that the integrated process improved anaerobic digestion in terms of volatile solids reduction, biogas production and reactor stability^6^,^7^,^8^.

Since the integration of multiple biological technologies shows positive effects on the degradation of digestate, a specific reactor layout should be designed to carry out such novel process. The anaerobic baffled reactor (ABR) shows the potential because it has already been successfully applied to many wastewater treatment plants due to its effective chemical oxygen demand (COD) removal, high tolerance towards loading shock, and capability to contain various biological metabolism phases^9^,^10^,^11^.

The ABR was initially developed by McCarty and coworkers^12^. A traditional ABR consists of a series of vertical baffles which force the wastewater flow under and over them as it passes from the inlet to the outlet^13^. Most importantly, the ABR is able to separate acidogenesis and methanogenesis horizontally along the reactor, thereby allowing the reactor to behave as a two-phase system^14^. This multi-compartmental structure encourages different bacterial groups to develop under their most favorable conditions, and enables the ABR to maintain a high biomass concentration. However, the accumulation of toxic residual substrates in upstream compartments will result in the inhibition of bacterial growth and metabolism^15^,^16^,^17^. A deficient amount of substrates in the downstream compartments could also lead to reduction of the reaction rate^18^,^19^. The degradation efficiency of organics is usually lower than that of aerobic processes since the traditional ABR process is operated under anaerobic conditions.

Microorganisms play an important role in wastewater treatment, and understanding the microbial community structure is of great importance for improving reactor performance^20^. Recently, the PCR-denaturing gradient gel electrophoresis (PCR-DGGE) of 16S rRNA genes has been used to rapidly monitor shifts in microbial community compositions^21^,^22^. The ABR, designed to achieve the separation of acid and methanogenic microbes with the best activity, was characterized by microbial alternation in different compartments along the flow direction^23^. Nachaiyasit and Stukey found that most microbes in the first compartment were butyric acid-producing bacteria, and *Methanogens* were dominant in downstream compartments^24^. Peng *et al*. investigated the spatial succession of functional microbial communities in a five-compartment ABR. The results showed that the acidogenesis stage and acetogenesis stage were located in the first two compartments, where H_2_-producing acetogens (19.7%) and H_2_-utilizing acetogens (8.3%) were the dominant bacteria. However, cloning and 16S rRNA gene analyses of the integrated system where the anaerobic and aerobic populations co-existed are still limited^25^.

In this study, a novel anaerobic/oxic baffled reactor (A/OBR) process was developed by adding a series of oxic compartments to a traditional ABR to enhance biodegradation. Operational parameters and various feeding strategies were investigated to reduce excessive loading shock, to optimize the distribution of substrate, and to improve COD removal. The objectives of this work were to evaluate the performance of the A/OBR, identify the optimal feeding strategy, and analyze its hydrodynamic characteristics. In addition, the development and composition of the bacterial community structure based on PCR-DGGE analysis were also determined to evaluate the process performance.

## Results

### Efficient COD removal by the novel A/OBR

A novel A/OBR was specially designed based on traditional ABR for the treatment of high salt and low pH foodwaste anaerobic digestate (Table 1). Three stages were divided artificially during the operation process according to the reactor and sludge characteristics. Stage I (day 1~14), the start-up stage, where microbes began to adapt to the environment and the characteristics of the reactors were quite unstable. The biomass increased with the increase of time and small granular particles or flocs formed. Stage II (day 15~28), the steady stage, where the reactor operation was relatively stable and granular sludge formed. The biomass concentration reached its maximum and a variety of bacteria were dominant in different compartments. Stage III (day 29~35), the final stage, where the operation system might be fluctuated or remain stable. Some of the dominant bacteria might fade and new dominant bacteria might appear.

**Figure 1:**
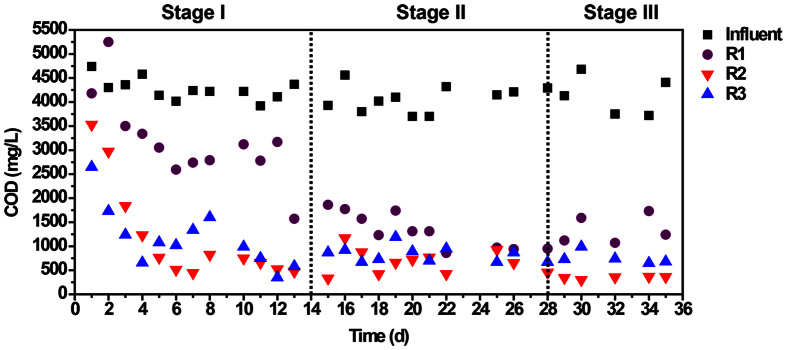
Influent and effluent COD concentrations of all A/OBRs at different stages. (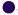)R1: regular-feeding; (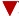) R2: split-feeding at ratio of 6:3:1, and (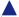) R3: split-feeding at ratio of 6:2:2. All three A/OBRs were domesticated for one month before the experiments and were operated in the same conditions.

The influent and effluent COD concentrations of the A/OBRs at different stages are illustrated in Fig. 1. Successful start-up of the A/OBR using regular-feeding strategy (R1) had been achieved during a 14-day operation at stage I, while the start-up time of both two split-feeding strategies (R2 and R3) was decreased to about 7 days due to the relief of the organic loading rate (OLR) in the upstream compartments. After the start-up, the operation of the three reactors was relatively stable at stage II. However, the COD removal in R1 at stage III became unstable due to the high OLR and some sludge bulking appeared, while that of R2 and R3 remained stable and efficient.

The average effluent and COD removal at different stages in the three A/OBRs are summarized in Table 2. In our study, COD removals at stage II and stage III were only 68.7 ± 9.5% and 66.4 ± 6.5% respectively in R1 (Table 2). However, two split-feeding reactors (R2 and R3) showed better efficiency and less variation compared with R1. The average COD removals of R2 and R3 were 84.1 ± 6.9% and 78.7 ± 3.8% at stage II respectively and slightly increased at stage III (91.7 ± 0.7% and 82.1 ± 3.5%, respectively). Considering the fact that the high variations of feedstock might cause fluctuation of COD removal, the one-way analyses of variance (ANOVA) were used to determine the significance of differences between reactors at different stages. The results showed that both two split-feeding reactors R2 and R3 could significantly improve the overall COD removal of stage I, II, and III (all *P* < 0.05) compared with regular-feeding reactor R1 (Table S1 in Supplementary Information). Among the three A/OBRs, R2 showed the best performance with the average effluent of 345 ± 27 mg/L and COD removal of 91.7 ± 0.7% at stage III (*P* < 0.05).

### Hydraulic characteristics of A/OBRs

**Figure 2:**
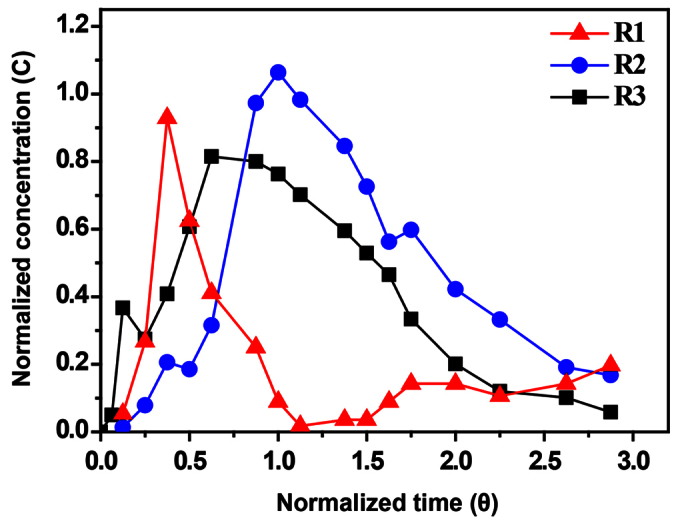
C-curves for the A/OBRs using regular-feeding and split-feeding strategies without biomass. Reactors were washed, filled with deionized water and then 0.56 mg K^+^/L was fed impulsively. Water samples of three reactors were collected at every 6 h intervals.

Residence time distribution (RTD) analysis was carried out to investigate the hydraulic characteristics of A/OBRs. Figure 2 showed the normalized concentration of K^+^ in the effluent against the normalized time (C-curve). The data from the C-curve were analyzed with a two-phase dispersion model and tanks-in-series (TIS) model^26^. Results are shown in Table 3.

#### Mixing patterns

Mixing patterns were analyzed by observing the variance of the C-curve. In the dispersion model, the findings were incorporated to calculate the dispersion number (*D*/*μL*)^27^. For *D*/*μL* above 0.2, the system is considered as completely-mixed, while *D*/*μL* below 0.02 it is considered as a plug-flow system^28^.

As shown in Table 3, dispersion numbers (*D*/*μL*) of two split-feeding strategies (6:3:1 and 6:2:2) were 0.14 and 0.15, respectively. These values were significantly lower than the *D*/*μL* of 0.33, which was calculated from the regular-feeding strategy. The results indicated that the mixing pattern in the regular-feeding reactor R1 was close to completely-mixed, while both two split-feeding reactors R2 and R3 were intermediate between plug-flow and completely-mixed, tending towards the latter.

In the TIS model, the reactor was characterized by a series of *N* equally sized CSTRs. When the value of *N* tends to ∞, the flow pattern of the reactor approaches plug-flow, and when *N* tends to 1, the reactor behaves as completely mixed^28^. Mixing patterns fitted by the TIS model showed the same trend as fitted by the dispersion model. However, the TIS model was the more useful since it could predict the degree of back-mixing in the reactor^26^. The larger the number of *N* in the reactor was, the smaller the amount of back-mixing that will occur^28^. Table 3 showed that the number of *N* in R1 was 1.35, indicating a large amount of back-mixing occurred in a regular-feeding strategy. However, both two split-feeding strategies showed significantly larger numbers of *N* (3.58 in R2 and 3.34 in R3 respectively). The larger number of baffles (*N*) inside the reactor inhibited back-mixing between compartments, although each individual compartment would be well-mixed. Therefore, mixing patterns of two split-feeding strategies were intermediate between plug-flow and completely-mixed, but closer to the completely-mixed pattern.

#### Hydraulic dead space

Dead space is divided into the categories of hydraulic dead space and biological dead space. The hydraulic dead space is a function of the flow rate and the number of compartments in the reactor while the biological dead space is a function of the biomass concentration and activity^26^. In order to better understand the effects of different feeding strategies on the hydraulic characteristics in A/OBRs, all three reactors were carried out without inoculation to eliminate the influence of biological dead space in RTD studies.

The evaluation of the hydraulic dead space mainly depends on the mean of the C-curve and the area under the curve between θ = 0 and θ = 2^26^. A lower dead space represents a better mixing in reactor. As shown in Table 3, the dead space in R1 was calculated as 31.9%. However, the dead spaces in R2 and R3 were 8.35% and 8.17%, respectively, which were significantly lower than that of R1.

### Bacterial community analyses by PCR-DGGE in A/OBRs

**Figure 3:**
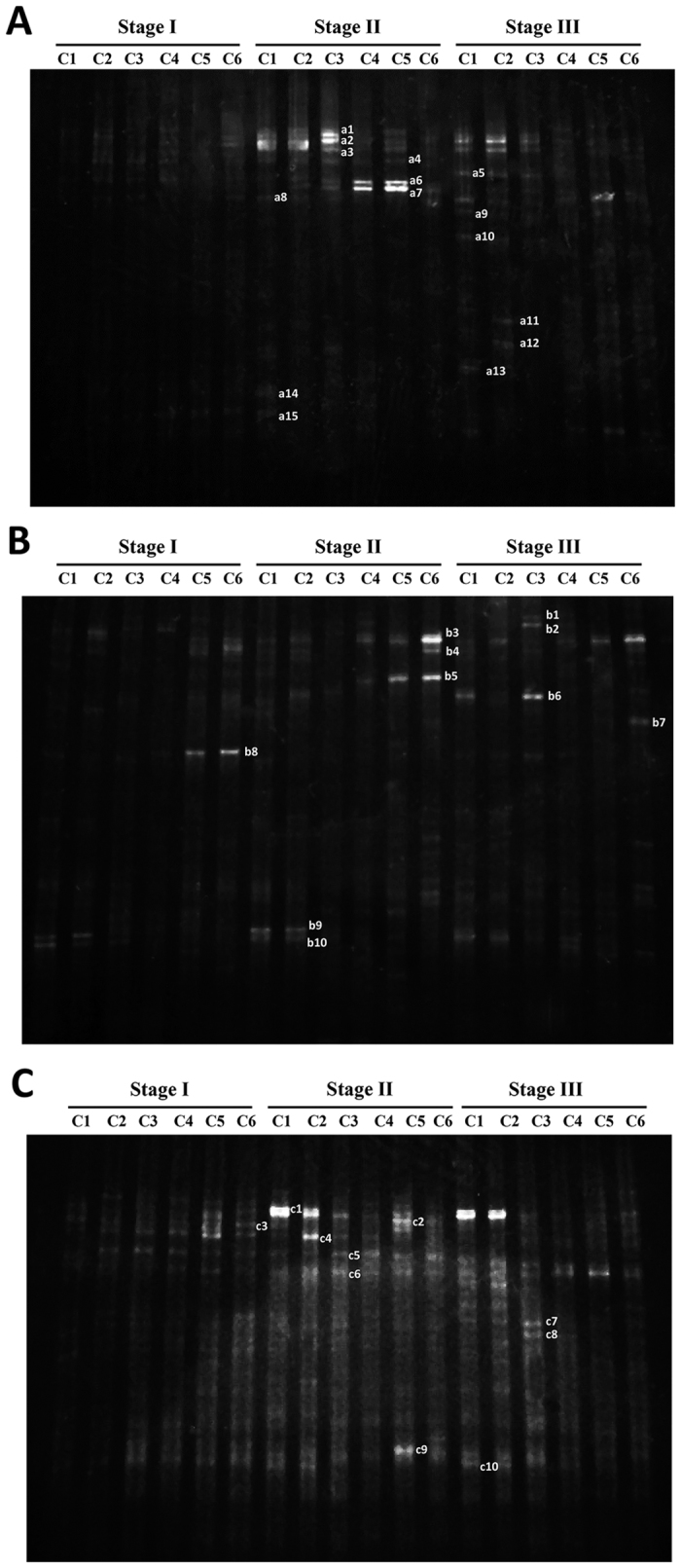
The DGGE profile for the bacterial communities analyses of (**A**) R1, (**B**) R2 and (**C**) R3 at different stages. Bands at the same horizontal position were the same species by further sequencing.

**Figure 4:**
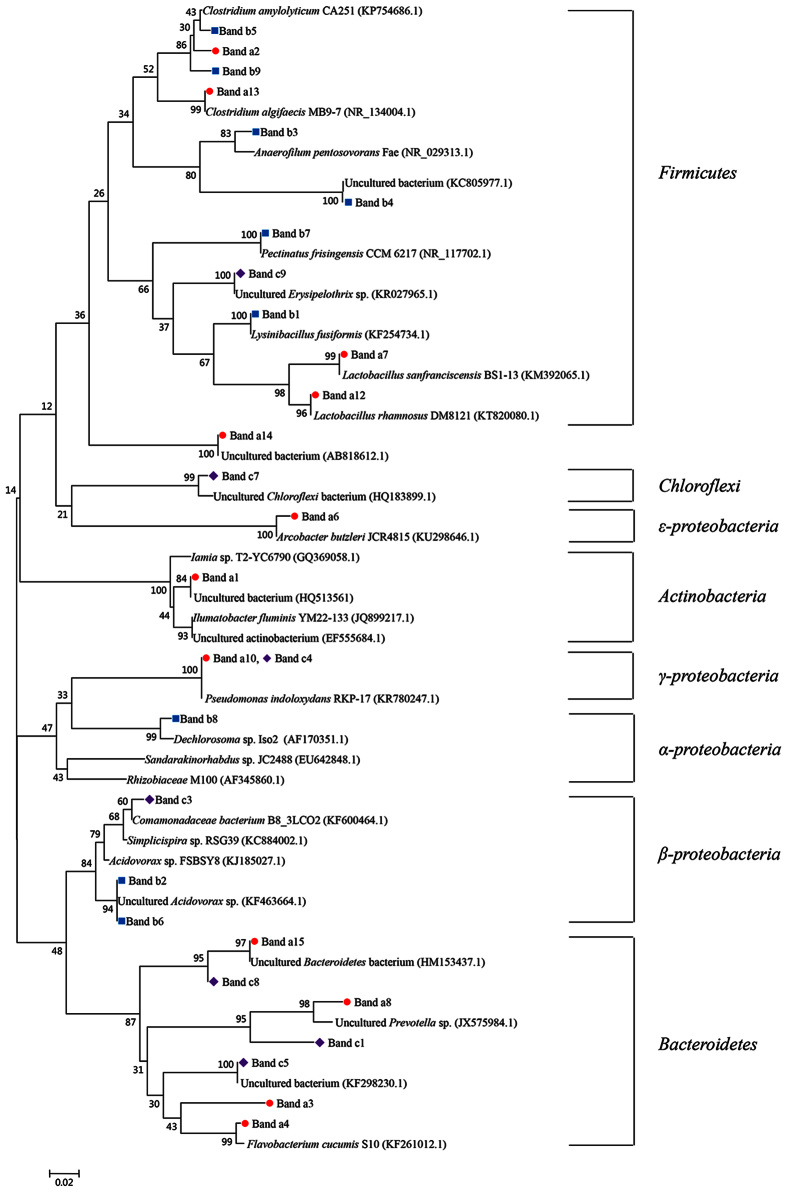
Neighbor-joining phylogenetic tree of 16S rRNA gene sequences from DGGE profile. Bands of (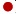) regular-feeding, (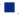) split-feeding at ratio of 6:3:1 and (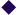) split-feeding at ratio of 6:2:2 at different stages were collected. Sequences were aligned using Clustal X 1.8 and MEGA 5.0 was used to construct phylogenetic tree. The bar represents 2% sequence divergence. The numbers in parentheses indicate GenBank accession number.

Samples from all compartments (C1–C6) in each reactor at different stages were taken, and the functional bacteria were analyzed by PCR-DGGE (Fig. 3). Twenty-nine predominant bands from DGGE gels of the anaerobic and aerobic compartments were sequenced for identification by BLAST analysis (Table 4) and the phylogenetic tree showing the phylogenetic identities of the 16S rRNA gene fragments was constructed (Fig. 4).

#### Spatio-temporal distribution of bacterial community

Generally, the distribution of bacterial composition in the A/OBR showed that most identified bacterial populations belonged to *Actinobacteria*, *Bacteroidetes*, *Chloroflexi*, *Firmicutes* and *Proteobacteria* (Table 4). This result was similar to previous studies of treating nitrobenzene^23^ and livestock wastewater^20^.

Bacterial community changes in the regular-feeding reactor were observed as shown in Fig. 3A. No dominant bands appeared at the start-up stage (stage I) while bright dominant bands were clearly observed in the fingerprints at stage II, which indicated that the microorganisms were gradually adapted to the environment and dominant bacteria formed. It was reported that the shift of community structure required some time to be visible because it would need time for microbes to fade out than to shut down their functional capability^20^. Bands a1, a2 and a3 were dominant in the first three compartments. Bands a6 and a7 were dominant in C4 and C5 at stage II, but decreased at stage III.

A community shift was also found in the split-feeding reactors (Fig. 3B,C). Band b5 was dominant in C4–C6 at stage II, especially in the aerobic compartment C5 and C6. Band b6 became dominant in C1 and C3 at stage III. Moreover, a new band b7 appeared in C6 at stage III (Fig. 3B). However, unlike for the regular-feeding strategy in A/OBR, most of the dominant bacteria in split-feeding at ratio of 6:3:1 were relatively stable with only a slight change at stage II and stage III, which indicated that the split-feeding strategies could promote the formation of a steady bacterial community. Similar trend in split-feeding at ratio of 6:2:2 could also be seen in Fig. 3C. Bands c1, c5 and c10 were quite stable and only band c4 in C2 disappeared at stage III.

#### Phylogenetic analysis

Though all reactors possessed almost the same dominant phylum, the detailed analysis showed significant differences in the terms of certain bacterial groups between the reactors (Fig. 4). Among a total of 29 bands of bacterial DGGE (Table 4), bands a2, a13, b5 and b9 belonged to *Clostridium* spp. Band b3, which was dominant in the downstream compartments (C4–C6) in split-feeding at ratio of 6:3:1, was closely related to *Anaerofilum* sp. These species were reported as acidogenic bacteria and could produce various organic acids as well as hydrogen^29^,^30^. Band b1 was identified as *Lysinibacillus* sp., which was a protein-fermentation-related genus due to its ability to secret α-chymotrypsin and oxidize various amino acids^31^,^32^. Some species of *Lysinibacillus* have been used in bioaugmentation to enhance anaerobic digestion of food wastewater^33^. Band a1 belonged to phylum *Actinobacteria*, most of which are heterotrophs, indicating that it might play an important role in reducing organic carbon^34^. Bands a8 and c1 were close to *Prevotella* sp., which was considered as a hydrogen-producing microorganism from organics and could also consume by-products such as acetate, succinate, or lactate^35^. *Flavobacterium* (band a4) as well as *Pseudomonas* were significant genera of flocforming bacteria due to its production of glue-like extracellular polymers and ability to bind cells together^36^. Besides, some denitrifying bacteria such as *Arcobacter* sp. (band a6), *Comamonadaceae* (band c3) *Pseudomonas* sp. (band a10 and c4), *Dechlorosoma* sp. (band b8) and *Acidovorax* sp. (band b2 and b6) were also found. Though bands a3, a14, a15, b4, c5, c7, and c8 could be classified as shown in Fig. 4, their functions are still unknown.

## Discussion

**Figure 5:**
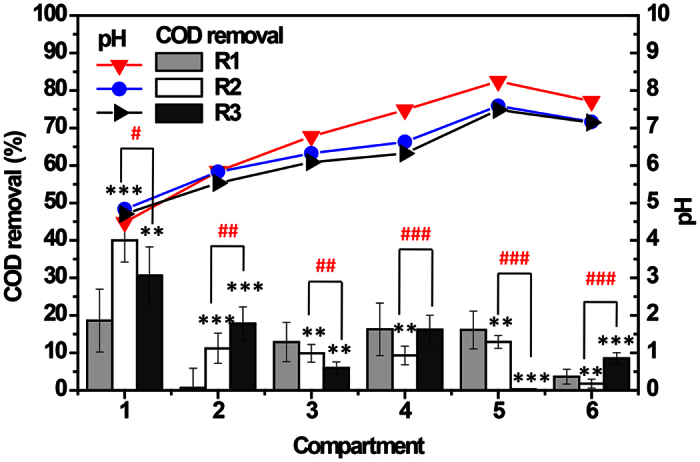
Average pH and COD removals for each compartment of all reactors at stage II. Average values and error bars were calculated from all daily data of stage II. The removal data for each compartment were calculated as: COD removal = (influent of this compartment – effluent of this compartment)/initial influent × 100%. Error bars represent SEM; *P* < 0.05 according to one-way ANOVA was considered significant and is indicated by *^/#;^ **^/##^*P* < 0.01; ***^/###^*P* < 0.001 (*Significantly different from R1, ^#^Significantly different between R2 and R3).

Many literatures have demonstrated that the first two compartments (C1 and C2) were crucial for the degradation of macromolecular organics and accounted for more than 50% COD removal in a ABR^37^,^38^. Nevertheless, excessive and/or toxic influent might greatly inhibit the degradation ability of C1 and C2. As a consequence, C1 and C2 only accounted for 19.3% (18.6% plus 0.7%) of the total COD removal in the regular-feeding reactor R1 in this study (Fig. 5). For this reason, C1 and C5 were changed into aerobic compartments to enhance the degradation ability, and a split-feeding strategy was employed to further reduce the inhibitions in the front compartments of the reactor. Consequently, the novel split-feeding A/OBR was established.

A pre-experiment was firstly conducted to determine the optimal feed ratio of C1, and three different feed ratios (70%, 60% and 50% of the initial influent that was fed into C1) were examined respectively. The results showed that the average COD removals in the last 7 days of the first two compartments were 38.3%, 51.4% and 43.2% respectively after a four-week operation (Table S2). This further indicated that C1 and C2 were still inhibited when 70% of influent was fed. On the other hand, less than 50% of the initial influent into C1 was meaningless though it might reduce the inhibitions, as the maximum removals of the first two compartments in theory were only 50%. Therefore, two split-feeding ratios (6:3:1 and 6:2:2) were chosen for this study.

Microbial growth and granule development were relatively lower in the anaerobic process than that in the aerobic process, and it usually took about 45~160 days to start-up in a traditional ABR^39^,^40^. However, the start-up period of the reactor would be greatly accelerated when a part of the anaerobic compartments were replaced by aerobic compartments. As shown in Fig. 1, the integration of aerobic and anaerobic processes in R1 could significantly shorten the start-up time to approximately 14 days. Moreover, the start-up time of two split-feeding reactors could be markedly reduced by 50%, which in turn led to the improvement in COD removal and stability of the A/OBR system. As summarized in Table 2, the COD removal in R1 was only 66.4 ± 6.5% at the end of the operation, which might be caused by the inhibition of excessive organic loading and high level toxic substances in upstream compartments. Yang *et al*. reported that it would be toxic to uncultivated microorganisms if the salt concentration in wastewater exceeded 1.0%^41^. Ji *et al*. used an ABR to treat heavy oil polluted water with high concentration of salt (1.15~1.46%), but the average COD removal was only 65%^42^. In our study, the high salt concentration (1.35~1.45%) might lead to a low COD removal in R1. Besides, there might be some other undetectable toxic compounds that could inhibit the microbes in the reactor due to the complex and fluctuant composition of foodwaste digestate. Therefore, a further increase in degradation efficiency could be expected after sharing the OLR and toxic substrate into the downstream of the reactor. As a result, COD removals of R2 and R3 increased to 91.7 ± 0.7% and 82.1 ± 5.5% respectively at stage III.

The ammonia-nitrite-nitrate conversion in three reactors at stage II was further analyzed and shown in Figures S1 and S2. Ammonium would be produced from the proteolysis of the proteinaceous substrates in the upstream compartments (C1–C3) and be assimilated by bacteria for amino acid synthesis. Excess ammonium then passed through downstream compartments and was utilized by denitrifying bacteria, resulting in a decrease in ammonium concentration (Figure S1)^43^. Figure S2 showed that denitrification occurred almost in the first compartment of all three reactors. Notably, ammonia increased in C5 with a slight decrease in nitrite/nitrate, indicating nitrification and denitrification process might occur simultaneously. Nevertheless, similar variations of ammonia, nitrite/nitrate were observed among three A/OBRs (Figures S1 and S2), resulting in a similar trend of pH (Fig. 5). Therefore, these factors might not be the main reasons for three reactors showing different COD removal efficiencies.

The average volatile fatty acid (VFAs) concentrations for each compartment of all reactors at stage II were shown in Figure S3. In a traditional ABR, most of the substrates were degraded into VFAs in upstream compartments, which were then utilized by methanogens to produce CH_4_ and CO_2_ in downstream compartments^44^,^45^. Similar trends could also be found in all three A/OBRs at stage II (Figure S3). Besides, Figure S3 showed that the VFAs of each compartment in R2 and R3 were relatively lower than that in R1 (all *P* < 0.05). This further demonstrated that the split-feeding strategy could relieve the OLR and toxic substrates for microbes in the upstream compartments and split it into downstream compartments, thus appearing as an increase in COD removal and decrease in start-up time.

As mentioned above, most of COD was degraded in the front compartments of the A/OBR, especially at relatively low organic loading rate (OLR). Polprasert *et al*. reported that COD was removed in the first compartment at an OLR of 0.87 kg/m^3^d. However, when the OLR increased initially to 1.82 kg/m^3^d and then to 4.73 kg/m^3^d, the larger fraction of COD was degraded both in the first and second compartments^46^. Manariotis and Grigoropoulos used an ABR to treat low-strength wastewater and found that most organic matter was degraded in the first two compartments, as the COD removals in the first three compartments were measured as 56.1%, 22.4% and 5.3%, respectively^47^. However, COD removal in each compartment was obviously different, though no apparent difference between R2 and R3 was observed in terms of total COD removal at stage II. In regular-feeding reactor R1, the first two compartments C1 and C2 only accounted for 19.3% (18.6% plus 0.7%) of the total COD removal, while it significantly increased to 51.2% (40.0% plus 11.2%, *P* < 0.05) and 48.5% (30.7% plus 17.8%, *P* < 0.05) in two split-feeding reactors R2 and R3 (Fig. 5). It should be highlighted that the ratio to the downstream compartments was important and an excess feed might reduce the COD removal ability. As shown in Fig. 5, COD removals in the last two compartments were totally different between R2 and R3 (*P* < 0.001). In R2, COD removals of C5 and C6 were 13.0% and 1.8%, while that in R3 were 0.3% and 8.5%, respectively. This difference might be caused by the high feeding rate to C5 in R3 (twice as much as that in R2), which would result in shorter retention time compared with R2. Therefore, there was no sufficient time for efficient COD removal. However, with the supplement of substrate from the C5 into the C6, more abundant microorganisms appeared in C6, thus showing a better removal.

Mixing patterns of the three A/OBRs were predicted by observing the variance in C-curve (Fig. 2). Date were then fitted into the two-phase dispersion model and the tank-in-series (TIS) model and results were summarized in Table 3. The results fitted by two models showed the same trend that the mixing pattern in the regular-feeding R1 was close to completely-mixed, while both two split-feeding reactors R2 and R3 were intermediate between plug-flow and completely-mixed, tending towards the latter. Moreover, split-feeding reactors R2 and R3 showed significantly advantage over regular-feeding reactor R1 in term of hydraulic dead space (8.35% and 8.17% versus 31.9%). Furthermore, it was reported that an increase in the (hydraulic) dead space was expected with a decrease in HRT because of more channeling in the reactor bed at high HRT (>20 h)^26^. Consistent with this conclusion, the calculated HRT in R1 was 44.6 h, which was much lower than that in R2 (57.2 h) and R3 (53.4 h), indicating that a significant amount of by-pass channeling took place in R1. Correspondingly, the fraction of dead spaces in R2 and R3 were much lower. Considering the fact that all three reactors were identical and operated in the same conditions except for the feeding ratios, it was reasonable to conclude that the split-feeding strategy could prevent channeling from taking place by creating a greater degree of fluidization in the reactor, and thus decreased the fraction of dead space.

In order to further study the function changes of different compartments, the diversity of bacterial communities determined by the Shannon-Wiener index (*H*’) is shown in Table 5. The Shannon-Wiener diversity index reflects the variety of bacterial communities and lower *H*’ values represents a lower α-diversity^48^. Table 5 showed that the *H*’ in R1 at stage II decreased constantly from C1 to C4, and then increased gradually in the last two compartments. As previously stated, the high OLR as well as high salinity inhibited the growth of microorganisms in upstream compartments, as reflected in a decrease in *H*’ value. On the contrary, *H*’ in R2 and R3 increased from C1 to C4, but decreased in the last two compartments. Compared to the R1, *H*’ values in R2 and R3 were relatively higher with less variation, which again provided the evidence that the split-feeding strategy could relieve the OLR and enhanced the degradation efficiency. Noticeably, the lowest *H*’ value of R3 was observed in C5, which agreed well with the above result that almost zero COD removal was obtained in C5.

Further phylogenetic analysis indicated that the most important organic matter degradation bacteria belonged to *Firmicutes*, *Bacteroidetes* as well as some of *Actinobacteria*, whilst the *Proteobacteria* played a crucial role in nitrification and denitrification. It has been proved that many *Clostridium* spp. such as *Clostridium aceticum*, *Clostridium formicoaceticum*, *Clostridium thermoaceticum* and *Clostridium cylindrosporum* harbored H_2_-utilizing ability and dominated in the acidogenesis/acetogenesis stage^25^. Aydina *et al*.^49^ also found that *Clostridium* spp. represented 93% of *Firmicutes* members in the seed sludge. Ren *et al*.^20^ indicated that *Firmicutes* was the most predominant and was more abundant in samples performing high COD removal efficiency. These dominant bacteria were relatively stable and played an important part in COD removal. Notably, some strictly anaerobic bacteria such as *Clostridium* sp. (band a2, a13, b5 and b9) and *Anaerofilum* sp. (band b3) were observed and even represented a major component in aerobic compartments C1 and C5, which may contribute to the formation of granular sludge. Similar results were also obtained in other aerobic reactors such as SBR and UASB^50^,^51^. This granulation allowed the gradient distribution of oxygen, thus making it possible for various aerobic, anoxic and anaerobic bacteria to be distributed throughout the granule layers. Moreover, this spatial distribution promoted the mass transfer to a certain extent and allowed nitrification and denitrification process occur simultaneously^52^. Figure 4 showed that *Arcobacter* sp. (band a6), *Comamonadaceae* (band c3) *Pseudomonas* sp. (band a10 and c4), *Dechlorosoma* sp. (band b8) and *Acidovorax* sp. (band b2 and b6) were denitrifying bacteria, which could use organic acids as electron donors and nitrate as an electron acceptor for denitrification^53^,^54^,^55^. Some of these denitrifying bacteria were also proven to have the anammox ability^56^, which might support the above inference that nitrification and denitrification might occur simultaneously in A/OBR.

It should be noticed that although PCR-DGGE was an excellent, highly reproducible, comparative community analysis tool^57^,^58^,^59^,^60^, the V3 region of the 16S rRNA might limit the amount of sequence information for precise identification. Therefore, only the genus level of bacteria was discussed in this study. Other nucleic sequencing method for cultivation-independent community analysis (e.g., 454 pyrosequencing, Illumina sequencing and metagenomic approaches) can be used in future research to develop a mechanistic understanding of the relationships between reactor operational strategies, microbial community structure, and reactor performance.

In summary, a novel, quick start-up and efficient anaerobic/oxic baffled reactor (A/OBR) was developed by incorporating an aerobic and anaerobic process for treating foodwaste anaerobic digestate. An effective split-feeding strategy was moreover presented to enhance the performance of A/OBR. Three aspects, the COD removal, the hydraulic characteristics and the bacterial community, were systematically investigated, compared and evaluated between the regular-feeding and split-feeding strategy. Compared with the regular-feeding reactor R1, the two split-feeding reactors R2 and R3 could greatly shorten the start-up time (~7 days), enhance the COD removal ability (more than 25%), reduce the hydraulic dead space (more than 20%) and maintain a more stable bacterial community. These characters implied that this new split-feeding A/OBR system might provide an effective and sustainable solution for treating foodwaste anaerobic digestate and other high COD and toxic wastewater. However, the feeding ratio should be tested to achieve the maximal removal efficiency before the application of this new system. Besides, some other wastewater treatment systems such as membrane bio-reactor (MBR), could also be combined with this A/OBR system to further enhance the treatment capability.

## Materials and Methods

### Reactor design and operation

**Figure 6:**
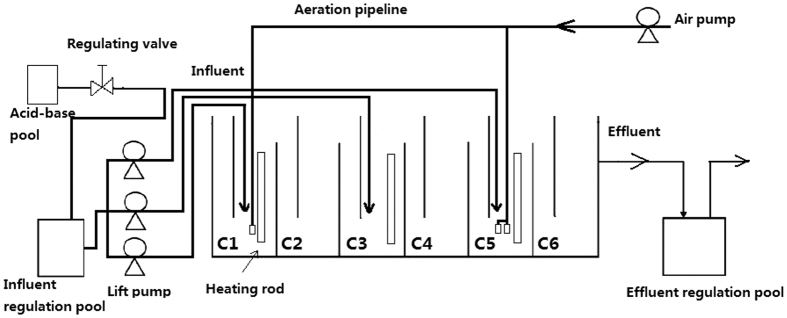
Schematic diagram of anaerobic/oxic baffled reactor (A/OBR) used in the experiments. Three peristaltic pumps were used to supply feedstock to C1, C2 and C5 at a total flow rate of 0.37 L/h. The flow rates provided by each pump were calculated according to the feeding ratios in different feeding strategies. All three reactors were carried out as calculated HRT of 48 hours under constant temperature of 35 °C.

The anaerobic/oxic baffled reactor (A/OBR), with dimensions of 600 mm long, 200 mm wide, 200 mm high, and an effective volume of 17.88 L, were used in the present study (Fig. 6). Each reactor was comprised of six compartments marked as C1, C2, C3, C4, C5 and C6, while each compartment was sub-divided by a vertical baffle into down-flow and up-flow sections with a volume ratio of 1:4. In order to increase the degradation efficiency of the reactor, an air diffuser was sunk into C1 to provide an oxic-anoxic-anaerobic-anaerobic condition for C1–C4. The main function of C1 and C2 was to decompose the macromolecular organic matter (such as starch, fat and protein) into small molecular substances (such as VFAs and amino acids). These small molecular substances were then converted to CH_4_, CO_2_ and H_2_ by anaerobic bacterium in C3 and C4. C5 was designed to further remove the residual organics by adding double air diffusers and C6 was designed as a settling compartment. Each air diffuser can provide an aeration rate of 1.25 L/min.

Three identical A/OBRs were employed to evaluate the effects of different feeding strategies in the present study. The regular-feeding strategy, in which the feedstock was loaded only into compartment C1 by one pump at the flow rate of 0.37 L/h, was tested in the first reactor (control group, labeled as R1). For the second and third reactors, two split-feeding strategies were carried out by splitting the total flow of 0.37 L/h into three streams and pumping into C1, C3 and C5 at volume ratio of 6:3:1 and 6:2:2, reactively (test group, labeled as R2 and R3). The purpose of split-feeding strategies was to relieve the loading shock in the first compartment. The hydraulic retention time (HRT) of the three reactors was 48 h. All three reactors were operated in the same conditions at 35 °C, except for the specifically applied feeding ratios.

### Characteristics of feedstock and inoculums

As feedstock for the present study, the discharged effluent was collected from a 100 m^3^ food waste anaerobic digester in the Changping district, Beijing. Samples were centrifuged at 5000 rpm for 5 min in a micro centrifuge (TGL-16G Centrifuge, Anting Scientific Apparatus Co., China). The pH was monitored by an ion meter (MP 523 pH/ISE meter, SANXIN Co., China). The COD, total nitrogen (TN), ammonia nitrogen (NH_4_^+^-N), nitrite (NO_2_^−^-N), nitrate (NO_3_^−^-N) and total phosphorus (TP) were analyzed according to the standard method^61^. The concentration of sodium was measured according to the standard method^50^ by atomic absorption spectrophotometer (SpectrAA55b, Varian, USA), as Chinese food usually contained high content of kitchen salt. The characteristics of the feedstock (Table 1) showed relatively high variations on element concentrations, which might be attributed to the feedstock contents and operation patterns of the food waste digester.

The A/OBRs were inoculated with dewatered anaerobic sludge collected from a wastewater treatment plant in the Shunyi District, Beijing, China. The inoculums had a VS/TS ratio of 0.32 and were initially fed into the empty reactors at a volume ratio of 30% of the reactor’s effective volume. All three reactors were operated under the same conditions after one month domestication before the experiments.

### Statistical analysis

Data analysis was performed with SigmaStat 3.5 and Excle. The one-way ANOVA were used to determine the significance of differences between groups, and *P* < 0.05 was considered as significant.

### Investigation of hydraulic characteristics

The hydraulic characteristics of the reactor were determined based on the residence time distribution (RTD) study by tracer stimulus-response technology^24^. The reactor was firstly filled with deionized water, and then KCl solution with concentration of 0.56 mg/L was fed intermittently. Water samples of three reactors were collected at every 6 h intervals. K^+^ concentrations were determined by atomic absorption spectrophotometer (SpectrAA55b, Varian, USA).

To compare the mixing patterns at different HRTs, the unit of time is normalized (dimensionless):


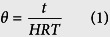


where, *θ* is the normalised time, *t* is the time. Effluent samples are collected at regular intervals from the time of impulse feeding to 3 times of nominal HRT (*θ* = 3), where the tracer concentration is too low to measure.


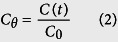


where, *C*_*θ*_ is the normalized tracer concentration at dimensionless time *θ*, *C(t)* is the tracer concentration at time *t*, *C*_*0*_ is the initial tracer concentration.

The C-curves (C vs θ), determined as a function of the normalized tracer concentration (Eq. (2)) against the normalized time (Eq. (1)), are shown in Fig. 2.

These curves were then further analyzed, as the calculated the mean (*θ*_*m*_) (Eq. (3)) and variance (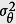) (Eq. (4)) of the curve, the fraction of dead space in the reactor (*V*_*d*_/*V*_*T*_), the overall dispersion number (*D*/*uL*), and the equivalence number of perfectly-mixed tanks in series (*N*)^26^.


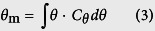



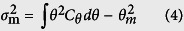


The fraction of dead space in the reactor was calculated using Eq. (5) as follows:


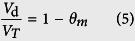


where, *V*_*d*_ is dead space and *V*_*T*_ is total volume.

In a dispersion model,


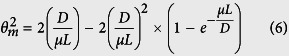


where, *D* is the axial dispersion coefficient, *μ* is the average fluid velocity and *L* is the axial distance of the reactor. *D*/*μL* is the reciprocal of the Peclet number *Pe*.

In a tank-in-series model,


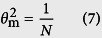


where, *N* is the number of on continuous stirred tanks in series.

### 16S rRNA gene amplification and denaturing gradient gel electrophoresis (DGGE)

The V3 region of the 16S rRNA gene was amplified by PCR using the primers 341F-GC (5′-CGC CCG CCG CGC GCG GCG GGC GGG GCG GGG GCA CGG GGG GCC TAC GGG AGG CAG CAG-3′) and 534R (5′-ATT ACC GCG GCT GCT GG-3′) for the bacteria^62^. The PCR protocol included 5 min pre-degeneration at 94 °C, followed by 30s denaturation for 30 cycles at the same temperature. Then the sample was annealed for 30s at 55 °C and then was extended for 30s at 72 °C, followed by 5 min additional extension at the same temperature. DGGE was performed on a DCode universal mutation detection system (Bio-Rad, USA). Samples of PCR products were loaded onto 8% polyacrylamide (Amresco, Ohio, United States) gels (37.5:1, acrylamide/bisacrylamide), and urea (Amresco, Ohio, United States) and deionized formamide (Amresco, Ohio, United States) (containing 7 mol/L urea and 40% formamide, defined as the denaturant concentration 100%) were added in one of the two solutions. The gel was prepared by using the Bio-Rad gradient mixing device (Bio-Rad, California, United States), to make the bacterial denaturant concentration range of approximately 40 to 65%, wherein the concentration of denaturant was decreasing from bottom to top. The sheet was put into a completely solidified electrophoresis tank containing 1× Tris-Acetate-EDTA buffer (Biotopped, Beijing, China), and the temperature was maintained at 60 °C during the whole electrophoresis. Pre-run was performed for 20 minutes under conditions of 180 V, in order to remove impurities in the gel. The 30 μL samples were loaded to the inlet. After electrophoresis at 180 V for 5.5 hours, the gel was stained for 30 minutes using a 3× GelRed (Biotium, California, United States) for further analysis. For identification of DGGE bands, each band was eluted into 40 μL of deionized and distilled water and then the mixture was incubated overnight at 4 °C to extract the DNA from DGGE bands. Solution was then used as the template in the reamplification reaction using the same primer without GC-lamp, the specific primers, 341F (5′-TAC GGG AGG CAG CAG-3′) and 534R (5′-ATT ACC GCG GCT GCT GG-3′)^63^.

The PCR products were sent to Hanyu biotech Co. Ltd. (Shanghai, China) for sequencing. The sequences were blasted with the nucleotide sequence databases in the GenBank and the BLAST program (http://www.ncbi.nlm.nih.gov/BLAST/). The migration and intensity of each band was analyzed using Quantity One 4.6.2 software (Bio-Rad, USA). The phylogenetic tree was constructed by MEGA version 5.1, using the neighbor-joining method. The Shannon-Wiener diversity index (*H*’) was calculated to evaluate the structural diversity of the bacterial communities by the following equation^64^:


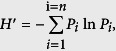


where *n* is the number of bands, and *P*_*i*_ is the relative intensity of the *i*_*th*_ band.

## Additional Information

**How to cite this article**: Wang, S. *et al*. Evaluation of A Novel Split-Feeding Anaerobic/Oxic Baffled Reactor (A/OBR) For Foodwaste Anaerobic Digestate: Performance, Modeling and Bacterial Community. *Sci. Rep.* **6**, 34640; doi: 10.1038/srep34640 (2016).

## Supplementary Material

Supplementary Information

## Figures and Tables

**Table 1:** Characteristics of the food waste digestate used as influent to the A/OBRs.

Parameter	Concentration*
COD	4250 ± 520 mg/L
Total nitrogen (TN)	110.8 ± 6.3 mg/L
Ammonia nitrogen (NH_4_-N)	30.2 ± 8.4mg/L
Total phosphor (TP)	8 ± 3 mg/L
NaCl	13.8 ± 0.6 g/L
pH	4.1 ± 0.5

^*^Average ± standard deviation for three independent samples.

**Table 2:** Average effluent and total COD removals at different stages in three A/OBRs.

ReactorNo.	Stage I		Stage II		Stage III	
	Effluent (mg/L)	Removal (%)	Effluent (mg/L)	Removal (%)	Effluent (mg/L)	Removal (%)
R1	3174 ± 898	32.8 ± 12.6	1281 ± 390	68.7 ± 9.5	1390 ± 267	66.4 ± 6.5
R2	1208 ± 1038	81.2 ± 10.4	653 ± 281	84.1 ± 6.9	345 ± 27	91.7 ± 0.7
R3	1178 ± 619	77.2 ± 8.9	872 ± 154	78.7 ± 3.8	740 ± 145	82.1 ± 3.5

R1: regular-feeding; R2: split-feeding at ratio of 6:3:1; R3: split-feeding at ratio of 6:2:2.

**Table 3:** Results from RTD analyses for three A/OBRs.

Reactor No.	HRT (Hour)	Variance of C-curve	*D/uL*^a^ (Dispersion model)	*N*^b^ (Tanks-in-series model)	Dead space (%)
R1	44.6	0.47	0.33	1.35	31.9
R2	57.2	0.28	0.14	3.58	8.2
R3	53.4	0.30	0.15	3.34	8.6

^a^Dispersion number (*D/uL*): a higher value represents the flow pattern is closer to completely-mixed.

^b^Tanks-in-series number (*N*): the number of theoretical stirred tanks, a higher value represents the flow pattern is closer to plug-flow.

**Table 4:** 16S rRNA gene sequence of DGGE bands in A/OBR.

Band No.*	Closest sequences	Identity (%)
a1	Uncultured bacterium	100
a2	*Clostridium* sp.	99
a3	Uncultured bacterium	93
a4	*Flavobacterium* sp.	98
a6	*Arcobacter* sp.	100
a7	*Lactobacillus* sp.	100
a8	Uncultured *Prevotella* sp.	98
a10	*Pseudomonas* sp.	100
a12	*Lactobacillus* sp.	99
a13	*Clostridium* sp.	99
a14	Uncultured bacterium	100
a15	Uncultured *Bacteroidetes* bacterium	100
b1	*Lysinibacillus* sp.	99
b2	Uncultured *Acidovorax* sp.	99
b3	*Anaerofilum* sp.	94
b4	Uncultured bacterium	100
b5	Uncultured *Clostridiales bacterium*	99
b6	Uncultured *Acidovorax* sp.	99
b7	*Pectinatus* sp.	100
b8	*Dechlorosoma* sp.	99
b9	*Clostridium* sp.	98
c1	Uncultured bacterium	100
c3	*Comamonadaceae* sp.	96
c4	*Pseudomonas* sp.	100
c5	Uncultured bacterium	100
c7	Uncultured *Chloroflexi* bacterium	99
c8	Uncultured *Bacteroidetes* bacterium	98
c9	Uncultured *Erysipelothrix* sp.	100

^*^Bands a5, a9, a11, b10, c2, c6 and c10 were not detected.

**Table 5:** Shannon-Wiener index of bacterial communities in different reactors at stage II.

Compartment No.	Shannon-Wiener index (*H*’)*		
	R1	R2	R3
C1	1.06	1.10	1.10
C2	0.92	1.18	1.12
C3	0.79	1.30	1.14
C4	0.73	1.30	1.20
C5	1.19	1.21	0.97
C6	1.17	1.27	1.12

^*^Shannon index (*H*’): a higher value represents more diversity.
