# Supplementary material for: Evaluation of A Novel Split-Feeding Anaerobic/Oxic Baffled Reactor (A/OBR) For Foodwaste Anaerobic Digestate: Performance, Modeling and Bacterial Community
Source: Sci Rep. 2016 Oct 6;6:34640. doi: 10.1038/srep34640 (PMC5052610; doi:10.1038/srep34640)
Supplement: Supplementary Information [file srep34640-s1.doc]

**Supplementary Information**

**Evaluation of A Novel Split-Feeding** **Anaerobic/Oxic Baffled Reactor (A/OBR) For Foodwaste Anaerobic Digestate: Performance, Modeling and Bacterial Community**

Shaojie Wang^1, +^, Liyu Peng^1, +^, Yixin Jiang^1^, Petros Gikas^2^, Baoning Zhu^3, *^ & Haijia Su^1, *^

^1^Beijing Key Laboratory of Chemical Resource Engineering, Beijing University of Chemical Technology, Beijing, 100029, PR China

^2^School of Environmental Engineering, Technical University of Crete, 73100, Chania, Greece

^3^Beijing Higher Institution Engineering Research Center of Environmental Pollution Control and Resource Utilization, Beijing University of Chemical Technology, No. 15 Beisanhuan East Road, Chaoyang District, Beijing 100029, PR China.

^*^Authors for correspondence: Suhj@mail.buct.edu.cn；bnzhu@mail.buct.edu.cn

^+^These authors contributed equally to this work.

Tel: +86-10-64453902

Fax: +86-10-64414268

**Table S1. One-way ANOVA analyses for the COD removals overall the stage I, II, and III.**

|  | **Stage I** | |  | **Stage II** | |  | **Stage III** | |
| --- | --- | --- | --- | --- | --- | --- | --- | --- |
|  | R2^a^ | R3^b^ |  | R2^a^ | R3^b^ |  | R2^a^ | R3^b^ |
| ***P^c^*** | 5.12E-05 | 0.00078 |  | 0.026 | 0.026 |  | 0.00049 | 0.0085 |

^a^The *P* values of R2 at all three stages were calculated by one-way ANOVA analysis using the data at different time points from R2 and R1.

^b^The *P* values of R2 at all three stages were calculated by one-way ANOVA analysis using the data at different time points from R3 and R1.

^c^*P* < 0.05 was considered significant

**Table S2. Average COD removals of first two compartments at different feeding ratios of C1.**

| **Compartment**  **No.** | **Average COD removal (%)^a,b^** | | |
| --- | --- | --- | --- |
|  | 70%^c^ | 60%^c^ | 50%^c^ |
| **C1** | 23.5 ± 4.6 | 39.4 ± 4.1 | 36.3 ± 2.1 |
| **C2** | 14.8 ± 3.2 | 12.0 ± 3.6 | 6.9 ± 2.5 |

^a^Average values and error bars were calculated from the daily data of last 7 days after four-week operation.

^b^The removal data for each compartment were calculated as: COD removal = (influent of this compartment – effluent of this compartment)/initial influent × 100%.

^c^The values represent the percentage of influent that was fed into C1.


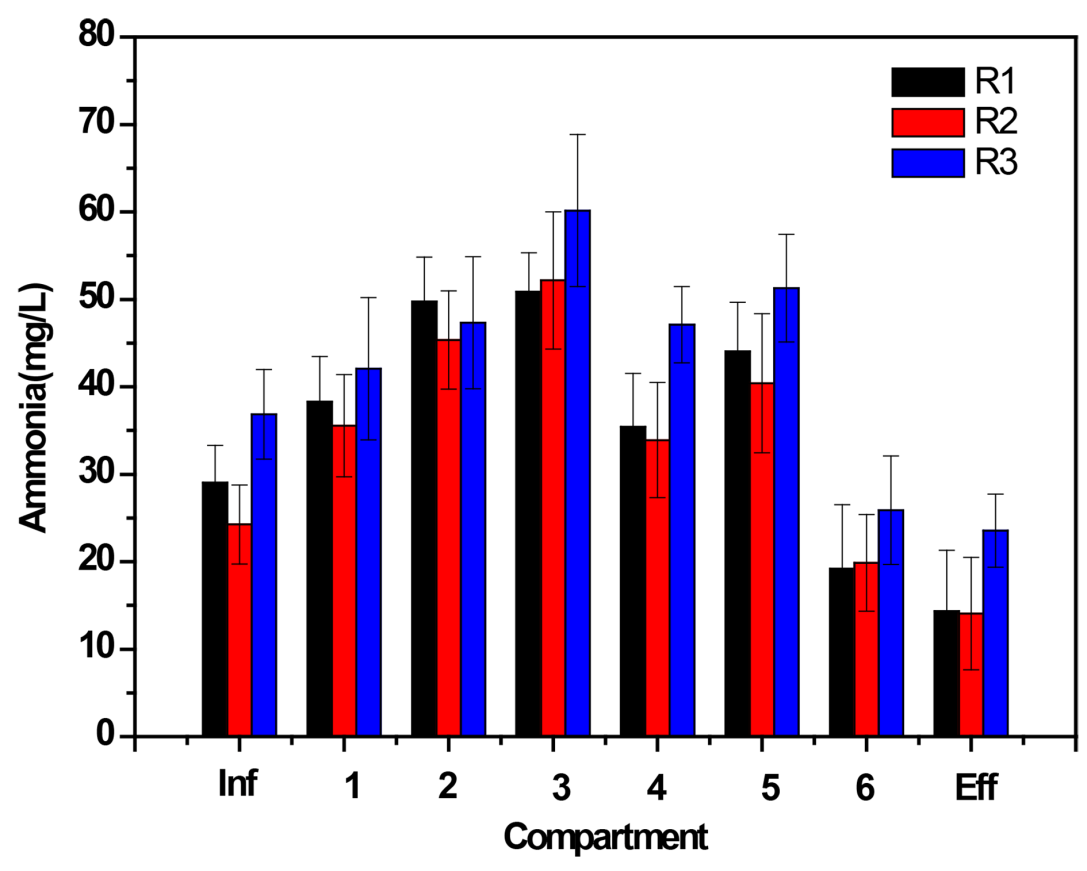


**Figure S1. Average ammonia for each compartment of all reactors at stage II.** Average values and error bars were calculated from all daily data in stage II (Inf: influent; Eff: effluent).


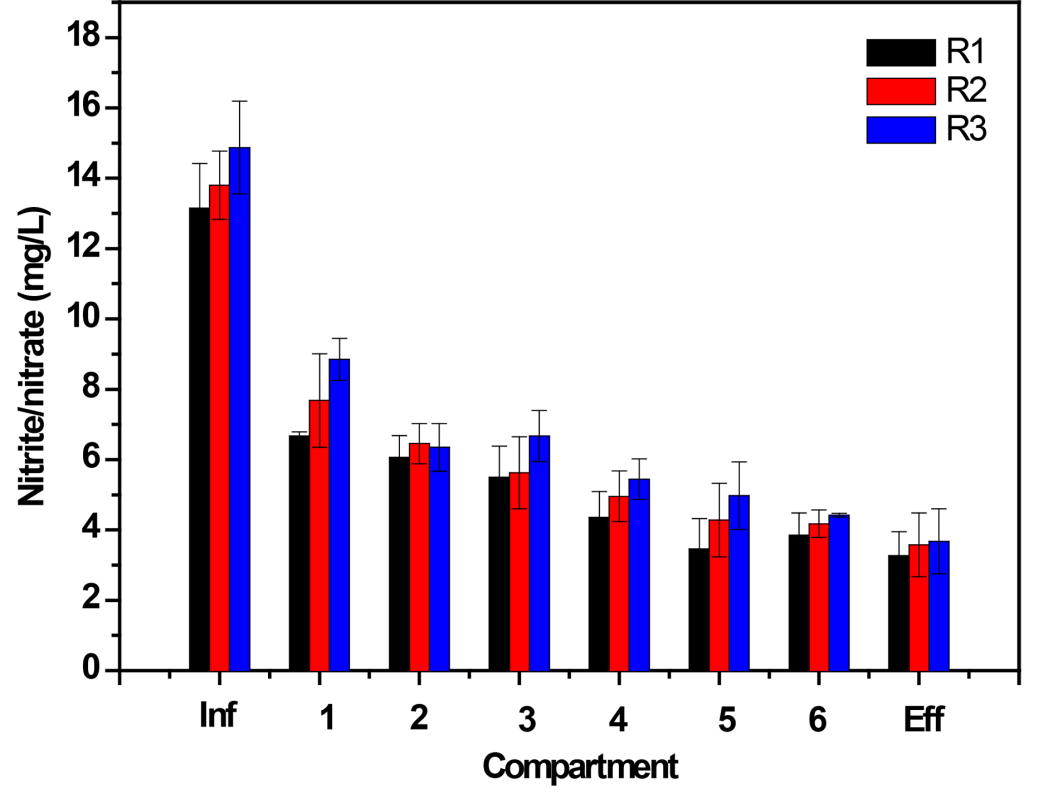


**Figure S2. Average nitrite/nitrate for each compartment of all reactors at stage II.** Average values and error bars were calculated from all daily data in stage II (Inf: influent; Eff: effluent).


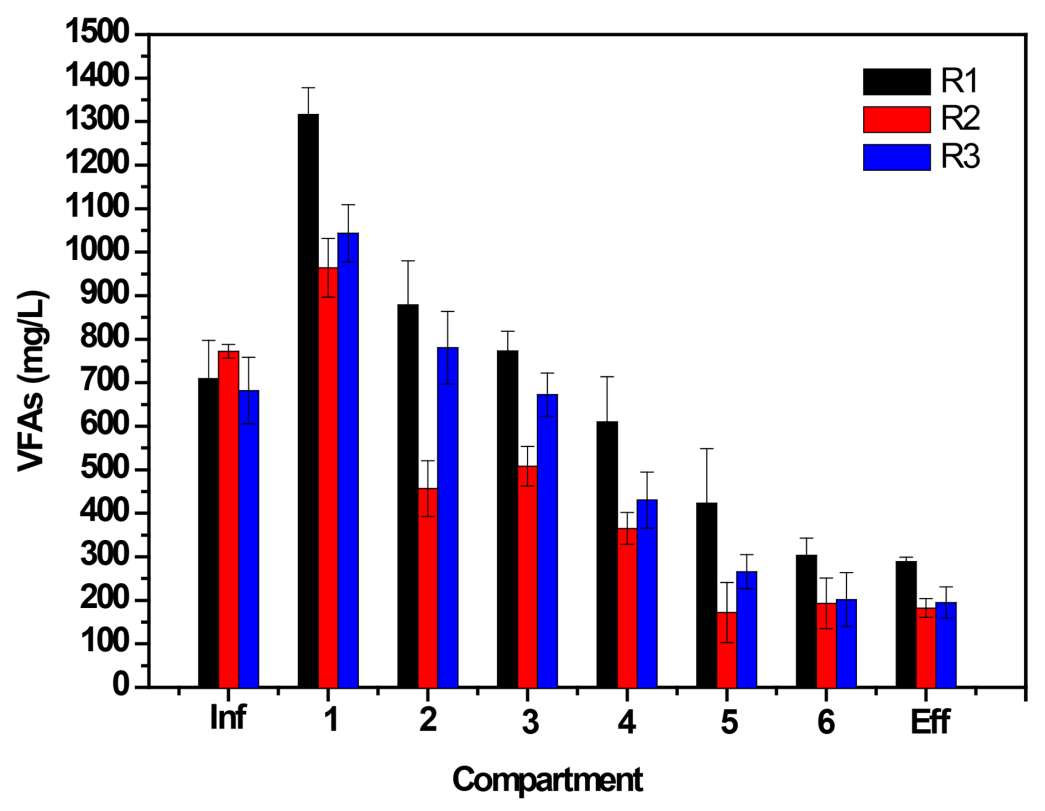


**Figure S3. Average VFAs for each compartment of all reactors at stage II.** Average values and error bars were calculated from all daily data in stage II (Inf: influent; Eff: effluent).
